# Identification of epigenetically regulated genes that predict patient outcome in neuroblastoma

**DOI:** 10.1186/1471-2407-11-66

**Published:** 2011-02-11

**Authors:** Helena Carén, Anna Djos, Maria Nethander, Rose-Marie Sjöberg, Per Kogner, Camilla Enström, Staffan Nilsson, Tommy Martinsson

**Affiliations:** 1 Department of Clinical Genetics, Institute of Biomedicine, University of Gothenburg, Sahlgrenska University Hospital, SE-413 45 Gothenburg, Sweden; 2 Genomics Core Facility, University of Gothenburg, SE-405 30 Gothenburg, Sweden; 3 Childhood Cancer Research Unit, Department of Woman and Child Health, Karolinska Institutet, Karolinska Hospital, SE-171 76 Stockholm, Sweden; 4 Molecular Medicine, Department of Medical Sciences, Uppsala University, SE-751 85 Uppsala, Sweden; 5 Department of Mathematical Statistics, Chalmers University of Technology, SE-412 96 Gothenburg, Sweden

## Abstract

**Background:**

Epigenetic mechanisms such as DNA methylation and histone modifications are important regulators of gene expression and are frequently involved in silencing tumor suppressor genes.

**Methods:**

In order to identify genes that are epigenetically regulated in neuroblastoma tumors, we treated four neuroblastoma cell lines with the demethylating agent 5-Aza-2'-deoxycytidine (5-Aza-dC) either separately or in conjunction with the histone deacetylase inhibitor trichostatin A (TSA). Expression was analyzed using whole-genome expression arrays to identify genes activated by the treatment. These data were then combined with data from genome-wide DNA methylation arrays to identify candidate genes silenced in neuroblastoma due to DNA methylation.

**Results:**

We present eight genes (*KRT19*, *PRKCDBP*, *SCNN1A*, *POU2F2*, *TGFBI*, *COL1A2*, *DHRS3 *and *DUSP23*) that are methylated in neuroblastoma, most of them not previously reported as such, some of which also distinguish between biological subsets of neuroblastoma tumors. Differential methylation was observed for the genes *SCNN1A *(p < 0.001), *PRKCDBP *(p < 0.001) and *KRT19 *(p < 0.01). Among these, the mRNA expression of *KRT19 *and *PRKCDBP *was significantly lower in patients that have died from the disease compared with patients with no evidence of disease (fold change -8.3, p = 0.01 for *KRT19 *and fold change -2.4, p = 0.04 for *PRKCDBP*).

**Conclusions:**

In our study, a low methylation frequency of *SCNN1A*, *PRKCDBP *and *KRT19 *is significantly associated with favorable outcome in neuroblastoma. It is likely that analysis of specific DNA methylation will be one of several methods in future patient therapy stratification protocols for treatment of childhood neuroblastomas.

## Background

Neuroblastoma (NB) is a pediatric tumor of the postganglionic sympathetic nervous system that develops from immature or de-differentiated neural crest-derived cells. It is the most common extracranial pediatric solid tumor, responsible for 15% of cancer deaths in childhood [1]. Much effort has been made to identify genes involved in the initiation/progression of NB. Tumor suppressor genes (TSGs) can be inactivated by mutations or deletions. Only infrequent mutations have been identified in neuroblastoma and homozygous deletions are rare events in primary NB tumors. Only a few have been reported in single cases [2-6]. Another mechanism for the inactivation of TSGs is epigenetic, where DNA methylation is the most studied. Several genes have been reported as being methylated in NB at different frequencies; for example the *RASSF1A *gene on chromosome arm 3p [7]. We have previously analyzed the methylation status of chromosome region 1p36 in NB, a region that is often heterozygously deleted in this tumor [8,9]. The regulation of gene expression also involves several modifications of histones. Acetylation and deacetylation on histones H3 and H4 are regulated by histone acetyltransferase (HAT) and histone deacetylase (HDAC). One approach to identifying candidate genes that are regulated epigenetically is to treat cancer cell lines with DNA methyltransferase inhibitors and/or histone deacetylase inhibitors, followed by an expression microarray analysis to identify genes that are upregulated by treatment. One advantage of this technique over direct methylation analysis is that it identifies epigenetic changes that also lead to a change in gene expression. One problem with this approach, however, is that several genes that are not methylated turn out to be positive for methylation, since these genes can be activated indirectly and not due to the demethylation of the respective gene. In this study, we have therefore combined a cell treatment study with a genome-wide DNA methylation analysis performed using the Illumina Human Methylation27 DNA analysis BeadChip to identify genes that are truly regulated by epigenetic mechanisms in NB cell lines and tumors. We selected a number of the identified genes and investigated them further; the selected genes were indeed methylated and the methylation frequencies of some of them were able to distinguish between different subgroups of NB tumors.

## Results

**Figure 1:**
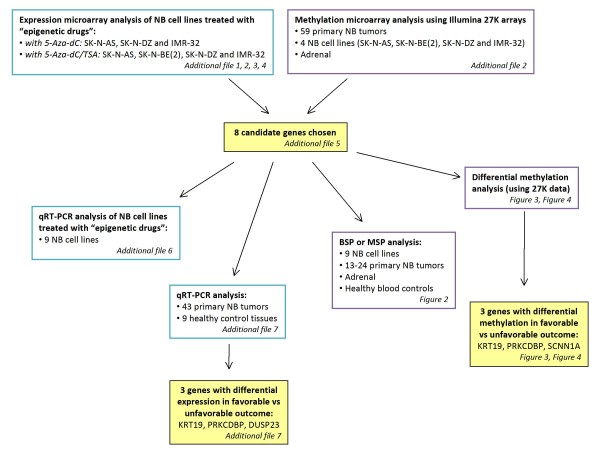
**Work flow of the analysis**. The figure describes the work flow of the analyses, samples used for respective analysis and gives a summary of all figures and additional files.

The work flow of the analysis including samples used is described in Figure 1.

### Validity of methods

Several genes previously known to be epigenetically regulated were identified in the 5-Aza-dC and/or TSA expression microarray treatment experiment. *ZMYND10 *(*BLU*), *THBS1 *and *SFN *(*14-3-3sigma*) have previously been reported to be methylated in NB [10-12]. Moreover, imprinted genes (for example, *NNAT *and *PEG3*) were detected, as expected. All these genes were also identified as methylated by the Illumina Human Methylation27 DNA analysis BeadChips analysis. Since the methylation status of all these genes is already known, they were not analyzed further. See additional file 1 and 2 for the number of genes affected in the treatments and additional file 3 for lists of the affected genes. The data from the Illumina Human Methylation27 DNA analysis BeadChips have been validated elsewhere (Carén *et al*, in manuscript).

The NB cell lines showed great heterogeneity in the cell treatment experiments (additional file 4). The largest number of upregulated genes after 5-Aza-dC/TSA treatment was found in SK-N-BE (1617 probes upregulated >2-fold) followed by IMR-32 (1422), SK-N-AS (1182) and SK-N-DZ (875). The same pattern was seen after only 5-Aza-dC treatment (IMR-32, 591 probes upregulated, SK-N-AS 334 and SK-N-DZ 84). The low number of genes upregulated by treatment in SK-N-DZ is consistent with the finding that SK-N-DZ has the lowest number of methylated CpG sites with a beta value above 0.75 (above half the number as the other cell lines).

### Selection of candidate genes

Genes were chosen as candidates for epigenetic regulation if they had a fold change of >2 between treated and untreated cell lines in at least one cell line. The common set of genes that was affected by both treatments was considered more likely to be true targets of epigenetic regulation and we therefore focused on this common set. Furthermore, genes that had no or only low expression in the untreated cell lines and that were affected by treatment, were considered as candidate genes. These data were combined with the Illumina methylation array data (Carén *et al*, in manuscript) and genes that also had a methylation beta value of more than 0.6 in primary NBs were selected. For the number of genes identified in the different steps, see additional file 2. The identified genes were examined for CpG islands using the UCSC genome browser and CpG island searcher. Ontology information was provided by the arrays; genes with functions related to cancer were selected. The gene lists were also used to search for NCBI abstracts using PubMatrix (URL: http://pubmatrix.grc.nia.nih.gov) with queries relating to neuroblastoma, methylation and tumorigenesis. After the bioinformatic analysis, nine genes were selected for further analysis, see Table 1 and additional file 5[13-37].

**Table 1:** Information of the genes studied in relation to tumorigenesis.

Symbol	Chr	Role	Methylated in other cancers	Comment
DHRS3	1p36	Short-chain dehydrogenases/reductase	Melanoma cell lines [17]	Enzyme involved in retinol metabolism. Putative TSG, crucial for the development of neural crest cells, located in NB SRO of deletions [16,18]. Recently reported as one of three candidate genes that were significantly overexpressed in favorable NB [19].
DUSP23	1q23	Dual-specificity protein phosphatase		Role in mitogenic signalling & cell cycle control. Fetal expression - possible role in early development [25,26].
TGFBI	5q31	Extracellular matrix protein	Leukemia, renal cell-, lung-, esophageal cancer [24]	Involved in cell adhesion and tumorigenesis [14,15]. Reduces proliferation and invasion *in vitro *and *in vivo *in NB. The mRNA expression of TGFBI is inversely correlated to MYCN expression in NB [19].
COL1A2	7q22	Type I collagen	Medulloblastoma, colorectal- breast- and bladder cancer, melanoma [13,21,23,29]	Tumors that secrete the gene have low tumorigeneic potential [27].
PRKCDBP	11p15	Protein kinase binding	Breast-, lung-, ovarian- and gastric cancer, glioblastoma multiforme [20,31,35-37]	Also called hSRBC. Putative TSG.
SCNN1A	12p13	Ion transport	Breast cancer [22]	Contributes to methylator phenotype in breast cancer [22].
POU2F2	19q13	Transcription factor		Regulator of neuronal differentiation [34].
KRT19	17q21	Intermediate filament protein	Renal cell carcinoma [30]	Involved in cell migration, invasion and metastasis [28]. Used as biomarker for detection of disseminated tumor cells [33]. Low expression in MNA NB [32].

TSG, tumor suppressor gene; NB, neuroblastoma; SRO, shortest region of overlap; MNA, *MYCN*-amplified

### Verification of the array data with expression analysis using real-time RT-PCR

The selected genes were analyzed in the same set and in another set of NB cell lines treated with 5-Aza-dC and/or TSA with real-time RT-PCR using TaqMan Technology. In most cases, there was a good correlation between the expression microarray data and the TaqMan data; when the gene was identified as upregulated by the array, it was generally also upregulated in the TaqMan analysis, although the magnitude of the values was not the same. Moreover, the new set of NB cell lines also showed upregulation of the genes after treatment (see additional file 6).

### Bisulfite sequencing and methylation-specific PCR

**Figure 2:**
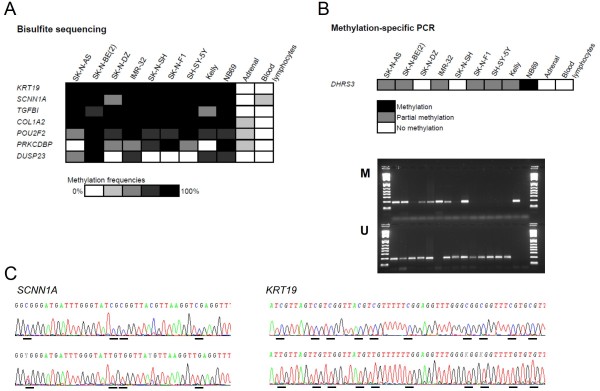
**Bisulfite sequencing analysis**. (A) Summary of bisulfite sequencing analysis of methylation status in cell lines and control tissues of the selected genes. (B) Methylation-specific PCR analysis of *DHRS3*. M, methylation-specific reaction; U, non-methylation-specific reaction. Samples from left to right: SK-N-AS, SK-N-BE(2), SK-N-DZ, SK-N-FI, Kelly, NB69, IMR-32, SK-N-SH, SH-SY-5Y, adrenal, blood lymphocytes (3 samples), unmethylated control, methylated control and negative PCR control. Most of the NB cell lines are partially methylated, as they show both methylated and unmethylated products. NB69 is although completely methylated, and SK-N-DZ and SK-N-SH unmethylated (although weak methylated bands can be detected by careful inspection, which could reflect a low level of methylation in these samples). (C) Examples of bisulfite sequencing of *SCNN1A *and *KRT19*. Cytosines/tymines in the CpG dinucleotide are underlined. C in the sequence indicates methylated CpG sites and T unmethylated. *SCNN1A*, top sequence IMR-32, bottom adrenal; *KRT19*, top sequence SH-SY-5Y and bottom, blood lymphocytes.

On the Illumina methylation arrays, the CpG sites can be located inside or outside of the CpG islands of the corresponding gene. In order to verify that the CpG site analyzed with the array was indeed methylated, or to ensure that, for the genes with the CpG sites outside the CpG islands, the corresponding CpG island was also methylated and to confirm that the surrounding CpG sites were methylated, bisulfite sequencing assays were designed. All genes were found to have methylated promoter CpG islands. The bisulfite sequencing analysis included DNA from NB cell lines, normal adrenal and blood lymphocytes from healthy blood donors for all the genes, as well as primary NB tumors (n = 13-34) for three of the genes (*SCNN1A*, *POU2F2 *and *COL1A2*). All the analyzed genes were unmethylated or showed low methylation in the adrenal sample, as well as in the blood lymphocyte samples. All the genes were methylated in more than half of the cell lines (Figure 2). The methylation beta value from the Illumina methylation arrays was compared with the peak heights of the sequencing electropherograms from bisulfite sequencing of the genes where the CpG site analyzed by the methylation array was inside of the PCR fragment analyzed by BSP (*TGFBI*, *PRKCDBP*, *DUSP23 *and *COL1A2*). There were good correlations between the methods.

### Differential methylation analysis

**Figure 3:**
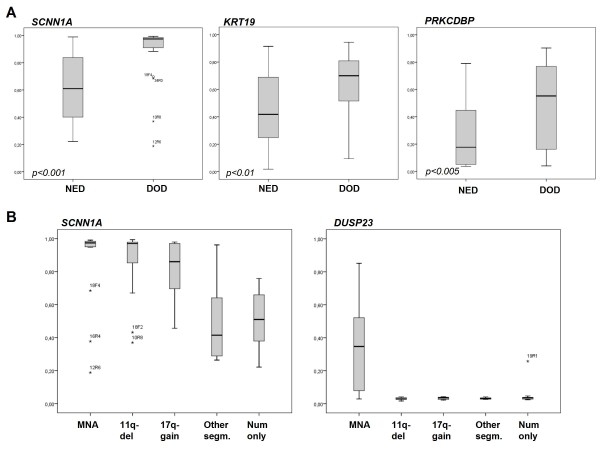
**Differential methylation analysis**. (A) Analysis of differences in methylation beta values from patients with no evidence of disease (NED) compared to those with an adverse outcome (DOD) for the most statistically significant genes. One of the CpG sites from the Illumina methylation array is shown. Box plot explanation; upper and lower hinges of the box represent the 75th percentile and 25th percentile respectively; whiskers indicate the highest and lowest values that are not outliers or extreme values; thick horizontal line within box, median. Open circles represent outliers and asterisks represent extremes. The p-value at gene-by-gene level is indicated in the left lower corner in each graph. (B) Analysis of methylation frequencies for tumors with different chromosomal profiles. For definition of chromosomal groups, see Carén *et al *[44]. CpG sites for *SCNN1A *and *DUSP23 *are shown. The highest methylation frequencies in *SCNN1A *are found in NB tumors with an unfavorable chromosomal profile. Methylation of *DUSP23 *is almost mutually exclusive in *MYCN*-amplified NBs.

In order to see whether the methylation also varied among biologically different subgroups of NB, we compared the methylation beta values from the Illumina arrays with different patient variables. We compared patients who are alive with no evidence of disease (NED) five years after diagnosis (5-year overall survival; OS) with those who were dead of disease (DOD), known prognostic chromosomal aberrations such as 1p deletion, *MYCN *amplification, 11q deletion and 17q gain, age at diagnosis, as well as chromosomal profiles obtained from array copy number data analysis [2]. Differential methylation based on 5-year OS was seen for the genes *SCNN1A*, *PRKCDBP *and *KRT19 *with a higher methylation frequency found in tumors from patients with an unfavorable outcome, see Figure 3A and Table 2. For *SCNN1A*, *PRKCDBP*, *KRT19*, *TGFBI *and *DUSP23*, significantly higher methylation frequencies found in *MYCN*-amplified tumors, see Figure 3B and Table 3.

**Table 2:** Methylation Δbeta values (methylation frequencies) between the outcome groups DOD and NED.

Gene	CpG site	In CpG island	Mean DOD (SD)	Mean NED (SD)	Δbeta value
SCNN1A	cg18738906	yes	0.88 (0.21)	0.65 (0.27)	0.23***
	cg26215727	no	0.47 (0.20)	0.31 (0.17)	0.16***
PRKCDBP	cg05628549	yes	0.53 (0.28)	0.34 (0.26)	0.18*
	cg16245261	yes	0.45 (0.34)	0.20 (0.23)	0.25**
	cg18392783	yes	0.50 (0.32)	0.26 (0.24)	0.24**
	cg18959478	yes	0.55 (0.31)	0.32 (0.27)	0.23**
KRT19	cg11462865	yes	0.65 (0.24)	0.43 (0.29)	0.22**
	cg16585619	no	0.82 (0.09)	0.81 (0.06)	0.01
TGFBI	cg00386408	yes	0.40 (0.24)	0.32 (0.23)	0.08
	cg21034676	yes	0.28 (0.28)	0.23 (0.24)	0.04
POU2F2	cg21608489	yes	0.62 (0.22)	0.59 (0.20)	0.03
	cg22054191	yes	0.22 (0.08)	0.24 (0.10)	-0.02
DUSP23	cg10663017	yes	0.20 (0.25)	0.11 (0.16)	0.09
	cg11104347	yes	0.17 (0.25)	0.10 (0.15)	0.08
DHRS3	cg01346152	yes	0.64 (0.30)	0.64 (0.26)	0.00
	cg09229231	yes	0.03 (0.01)	0.03 (0.01)	0.00
COL1A2	cg18511007	yes	0.42 (0.27)	0.35 (0.25)	0.07
	cg25300386	yes	0.37 (0.29)	0.32 (0.27)	0.05

DOD, dead of disease; NED, no evidence of disease; *p ≤ 0.05; **p < 0.01; ***p < 0.001; SD, standard deviation

**Table 3:** Methylation Δbeta values (methylation frequencies) between various tumor characteristics.

Gene	CpG site	INRG (M/L)	**1p-del**^**a**^	**MNA**^**a**^	**11q-del**^**a**^	**17q-gain**^**a**^	>18 m
SCNN1A	cg18738906	0.33***	0.15	0.21**	0.25***	0.34***	0.15*
	cg26215727	0.15**	0.04	0.13*	0.17**	0.20***	0.09
PRKCDBP	cg05628549	0.09	-0.06	0.12	0.08	0.09	-0.10
	cg16245261	0.17*	0.00	0.27***	0.15	0.19*	0.01
	cg18392783	0.12	-0.06	0.18**	0.10	0.12	-0.02
	cg18959478	0.12	-0.06	0.16*	0.10	0.11	-0.04
KRT19	cg11462865	0.14	-0.01	0.19*	0.10	0.12	0.22**
	cg16585619	0.01	-0.05*	0.07***	0.04*	0.03	0.01
TGFBI	cg00386408	0.05	-0.04	0.11	0.10	0.07	0.14*
	cg21034676	0.04	-0.04	0.13	0.06	0.05	0.12
POU2F2	cg21608489	-0.08	-0.05	-0.08	-0.01	-0.04	-0.08
	cg22054191	0.03	-0.03	-0.01	0.04	0.02	0.04
DUSP23	cg10663017	0.06	-0.10	0.27***	0.21***	0.16**	0.07
	cg11104347	0.05	-0.13*	0.29***	0.16**	0.13**	0.08
DHRS3	cg01346152	-0.13	-0.03	-0.20**	-0.03	-0.08	-0.10
	cg09229231	0.004*	0.00	0.00	0.00	0.00	0.00
COL1A2	cg18511007	0.07	0.00	0.05	0.07	-0.01	0.18**
	cg25300386	0.02	-0.04	-0.02	0.02	-0.09	0.15*

M, metastatic; L, localized; 1p-del, 1p-deletion; MNA, *MYCN *amplification; 11q-del, 11q-deletion; m, months; ^a^presence of chromosomal aberration tested against absence; *p ≤ 0.05; **p < 0.01; ***p < 0.001

The odds ratio (OR) for 5-year OS was calculated using *MYCN *amplification, 1p deletion, 11q deletion, 17q gain or gene methylation as prognostic factors. The OR was 5.7 for *MYCN *amplification (95% CI 1.7-19.1), 4.4 for 1p deletion (95% CI 1.4-13.6), 3.4 for 11q deletion (95% CI 1.1-11.1) and 10.8 for 17q gain (95% CI 3.0-38.7). For gene methylation, the ORs are given for one (1) standard deviation increase in beta units of methylation. The OR for *SCNN1A *was 3.4 (95% CI 1.5-7.3), for *PRKCDBP *2.5 (95% CI 1.3-4.8) and for *KRT19 *2.5 (95% CI 1.2-5.0). As a prognostic tool, known factors such as *MYCN *amplification predict the outcome of 70% of cases in our material correctly, 1p deletion 69%, 11q deletion 66% and 17q gain 74%. Using the estimated logistic regression model and a cut-off at 50% risk, the methylation beta value of *SCNN1A *predicts 77%, *PRKCDBP *76% and *KRT19 *64% of cases correctly.

### Expression analysis with real-time RT-PCR

Real-time RT-PCR data of the eight genes was generated from primary tumors, NB cell lines and control tissues using custom-designed TLDA cards (Applied Biosystems, Foster City, CA). All the genes were expressed in all control tissues. *SCNN1A *expression was absent in 44% of the NB tumors analyzed, the other genes were expressed in all tumors. The mRNA expression of the genes *KRT19*, *PRKCDBP *and *DUSP23 *was significant lower in patients that have died from disease compared with patients with no evidence of disease (fold change -8.3, p = 0.011, -2.4, p = 0.036 and -2.8, p = 0.017, respectively; see additional file 7).

## Discussion

In this study, we treated four NB cell lines with 5-Aza-dC and/or TSA and analyzed the RNA with expression microarrays. Data from Illumina genome-wide methylation arrays (Carén *et al*, in manuscript) of 59 primary NB tumors were also used to identify genes that were activated by treatment as well as being methylated. Eight genes were selected as candidate genes for epigenetic silencing and the data from the 5-Aza-dC and/or TSA expression microarray were validated for these genes with real-time RT-PCR in the same set of cell lines, as well as in five additional NB cell lines. The results from the microarray and the real-time RT-PCR analysis was generally in accordance and most of the additional five cell lines showed the same pattern of gene activation after 5-Aza-dC and/or TSA treatment (see additional file 6). Bisulfite sequencing was performed to validate the methylation status in the respective promoter CpG islands; all the eight genes were indeed methylated in the region surrounding the transcription start sites. Differential methylation beta values from the Illumina methylation arrays were analyzed to explore if the beta values of the eight genes could be used to distinguish different subsets of NB. See table 2 for various tumor characteristics that could be distinguished using methylation beta values of different genes. A high beta value was generally associated with unfavorable characteristics. For example, the beta values of *SCNN1A*, *PRKCDBP *and *KRT19 *could distinguish between patients with a 5-year OS from those that have died of the disease. The beta values of these three genes as well as for *TGFBI *and *DUSP23 *were also correlated with *MYCN *amplification. *TGFBI *expression has previously been shown to be inversely correlated to *MYCN *amplification [14]. Our data suggest that DNA methylation could be a factor that is responsible for this. Real-time RT-PCR analysis of the eight genes was also performed. Gene expression could be detected in most of the NB tumors and in all control tissues (*SCNN1A *was only expressed in 56% of NB tumors). The mRNA expression of *KRT19*, *PRKCDBP *and *DUSP23 *was significantly lower in tumors from patients that have died from the disease compared with patients with no evidence of disease (additional file 7). The beta value of the gene *KRT19 *(CpG site cg11462865) was correlated (by Spearman correlation; correlation -0.54, p = 0.002) with the mRNA expression in paired samples. Also for *DHRS3 *a correlation was detected (cg01346152, correlation -0.42, p = 0.02). The other genes did not show this paired sample correlation, indicating that the analyzed CpG sites in these genes are located in regions that are not important for gene regulation. There are also technical explanations for this. For example, since the DNA and RNA have been extracted using different samples of the same tumor the heterogeneity of the tumor could explain that correlations are not seen since it is not the identical sample that is analyzed. The fact that correlations in DNA methylation and mRNA expression are not seen for all the genes may also reflect the difficulties involved in analyzing RNA; RNA is much more unstable and degrades far more easily than DNA. In contrast, the methylation of DNA is a robust, stable procedure and much easier to measure. This could also explain why DNA methylation is far more closely correlated to various tumor characteristics than gene expression (see Table 2 and additional file 7).

The three identified genes with differential methylation (*SCNN1A*, *PRKCDBP *and *KRT19*) have all previously been reported as methylated in different forms of tumors. *SCNN1A *is an ion transport gene that has been reported to be one of six genes that contribute to a hypermethylator phenotype that is seen in a subset of breast cancer cell lines and primary tumors [22]. Hypermethylation of the putative tumor suppressor gene *PRKCDBP *(also known as *hSRBC*) has been reported in several different tumors (Table 1). Stable expression of *PRKCDBP *has been shown to induce cell cycle arrest in the G1 phase as well as apoptosis, and suppress cellular growth *in vitro *and in xenograft tumors by enhancing the protein stability of p53 and the expression of p53 target genes [20]. *KRT19 *(*CK19*) encodes an element of the cytoskeleton and shows frequent DNA methylation in renal cell carcinoma (RCC) cell lines and primary RCC, without methylation in normal renal tissue [38]. *KRT19 *mRNA has also been reported to be downregulated in squamous cell carcinoma (SCC) of the head and neck and the over-expression of the gene was shown to decrease SCC invasiveness by diminishing migratory capability [39].

## Conclusions

Based on our data, *SCNN1A*, *PRKCDBP *and *KRT19 *are the best candidates for further analysis in NB. The methylation frequencies of all of these genes can separate tumors from patients with no evidence of disease from those that have died from disease. The mRNA expression of *KRT19 *and *PRKCDBP *is also significantly different in tumors from these two groups of patients. It would therefore be very interesting to explore the functions of these genes in relation to NB development/progression and their use as potential biomarkers for prognostic use.

The number of tumors analyzed in this study was fairly small (n = 59) and the findings that the methylation pattern of the genes mentioned here is associated with different biological subgroups of NB need to be verified in a larger number of tumors. Nevertheless, as a prognostic tool, the methylation of most of the genes presented here is just as good or better as the known prognostic risk factors, such as 1p deletion, *MYCN *amplification, 11q deletion and 17q gain, when it comes to predicting accurate outcome in this set of tumors. In addition, this study uses a technique that has not previously been used for this tumor and it highlights genes with interesting functions that have not previously been reported as methylated in NB. Stable biomarkers as DNA methylation are likely to be important in the risk stratification of patients with NB in the future. It is essential to make the best possible prognosis in order to cure as many patients as possible, as well as to avoid unnecessary treatment which can lead to severe side-effects in this group of young patients.

## Methods

### Cell lines and tumor material

#### Expression analysis

The four NB cell lines SK-N-AS, SK-N-BE(2), SK-N-DZ and IMR-32 were used for cDNA microarray analysis. For conformation studies with real-time RT-PCR, these four and another five NB cell lines (SK-N-SH, SK-N-FI, SH-SY-5Y, Kelly and NB69) were used, as well as 43 NB tumors and nine healthy control tissues (fetal brain, fetal heart, fetal kidney, fetal spleen, fetal thymus, adrenal, colon, leukocytes and mammary gland; all from Clontech Laboratories, Mountain View, CA). Ethical permission was granted by the local ethics committee (Karolinska Institutet and Karolinska University Hospital, registration number 03-736 and 2009/1369).

#### Methylation analysis

Fifty-nine NB tumors were analyzed with the Illumina Human Methylation27 DNA analysis bead chips, together with one adrenal sample, methylated and unmethylated controls and four NB cell lines, SK-N-AS, SK-N-BE(2), SK-N-DZ and IMR-32 (additional file 8). Thirty-two of the tumors used for expression analysis were included in the methylation analysis. For bisulfite sequencing, nine NB cell lines were used, together with control DNA from healthy blood donors and one adrenal sample. Thirty of the tumors analyzed with the methylation arrays were also used to verify the methylation status of some of the selected genes. In addition, a methylated control sample and an unmethylated control (EpiTect control DNA, Qiagen, Hilden, Germany), as well as a 50/50 mixture of them, were used in the amplification of bisulfite-modified DNA, in order to control for the unwanted selective amplification of methylated or unmethylated templates during PCR amplification.

### Drug treatments

Cells were treated with the demethylating agent 5-Aza-2'-deoxycytidine (Sigma-Aldrich CO, St Louis, MO) or the histone deacetylase inhibitor trichostatin A (TSA; Sigma-Aldrich) or with a combinatorial treatment with both agents as previously described [9]. A concentration of 2 μM of 5-Aza-2'-deoxycytidine (5-Aza-dC) for 72 hours and 0.5 μM of TSA for 16 hours was used.

### RNA extraction

Total RNA was extracted from cell lines and primary tumors using the Totally RNA kit (Ambion, Austin, TX) and was treated with DNA-*free *(Ambion), according to the protocols of the supplier. RNA quality was assessed using an Agilent 2100 Bioanalyzer (Agilent, Palo Alto, CA) and by measuring absorbance with the Nanodrop ND-1000 (NanoDrop Technologies, Wilmington, DE).

### Expression microarray analysis

RNA was analyzed with the Human-6 v2 Expression BeadChip (Illumina Inc., San Diego, CA) at AROS Applied Biotechnology AS (Aros AB, Aarhus, Denmark), according to the protocol provided by the supplier. For the generation of biotin-labeled cRNA, the Illumina TotalPrep RNA amplification kit (Ambion) was used. In short, 300 ng of total RNA was reverse transcribed and the first-strand cDNA was used to make the second strand. The purified second-strand cDNA, along with biotin UTPs, was then transcribed *in vitro *into biotinylated cRNA. Purified, labeled cRNA, 1.5 μg, was hybridized to Sentrix Human-6 v2 expression Illumina Beadchips for 16 h at 58°C, before being washed and stained with streptavidin-Cy3. The bead chips were then dried and scanned on the Illumina BeadArray Reader confocal scanner. Expression data generated by BeadStudio were exported and analyzed using IlluminaGUI [40].

### Analysis of DNA methylation

Illumina Human Methylation27 DNA analysis bead chips were used to determine the methylation levels of 27,578 CpG sites. After bisulfite treatment of the DNA samples, the cytosines in the CpG sites were genotyped as C/T polymorphisms according to the manufacturer's protocol. The fluorescence signals were measured from the BeadArrays using an Illumina BeadStation GX scanner. The fluorescence data were then analyzed using the BeadStudio software (Illumina). The software assigns a score called a "beta value" to each CpG site, which corresponds to the ratio between the fluorescence signal from the methylated allele (C) and the sum of the fluorescent signals of the methylated (C) and unmethylated (T) alleles [41]. The array processing was performed by the SNP Technology Platform in Uppsala (URL: http://www.genotyping.se).

### cDNA preparation and real-time RT-PCR

cDNA preparation was performed as previously described [9]. Custom-designed TLDA cards containing 15 individual assays were ordered from Applied Biosystems. 200 ng of RNA converted into cDNA in a total volume of 100 μl were loaded to each card according to the instructions of the manufacturer. Each cDNA sample was analyzed in triplicate. TLDA cards were run and analyzed by the ABI PRISM^® ^7900HT Sequence Detection System (SDS 2.2, Applied Biosystems) according to manufacturer's protocol (Applied Biosystems). Calculations were performed using the ΔCt relative quantification method. The thresholds and baselines were set manually in SDS and Ct values were extracted. All Ct values were normalized to the housekeeping gene *GUSB *for each sample [9]. Samples with no expression were set to a Ct value of 40. To evaluate the agreement between the mRNA expression levels and the DNA methylation levels, a Spearman correlation coefficient was calculated for each gene. The delta Ct values between groups were compared using Student's two-sided t-test. Fold change between groups was calculated from the 2^-delta Ct values.

### DNA methylation analysis with bisulfite sequencing

CpG islands at the 5' promoter region that included the transcriptional start sites were identified using CpG island searcher (URL: http://cpgislands.usc.edu) [42]. These regions, or parts of them, were amplified with regular PCR or semi-nested/nested primers if needed. Primers were designed with BiSearch [43]. Primer sequences are available on request.

Methylation analysis was performed with tag-modified bisulfite genomic sequencing [44]. Genomic DNA, 1 μg, was modified with the EpiTect kit (Qiagen), according to the protocol of the supplier. The modified DNA was amplified using touchdown PCR with 1x Reaction Buffer, 0.5 mM dNTPs, 2.0-3.0 mM MgCl_2_, 0.4 μM of forward and reverse primers respectively and 1 unit of HotStar Taq (Qiagen), in a total volume of 20 μl, with or without the addition of Q-solution (Qiagen). Reactions were denatured at 95°C for 10 min, followed by 20 cycles of 95°C for 45 sec, 10°C above annealing temperature with a decrease of half a degree per cycle for 45 sec, 68°C for 60 sec and 15-20 cycles of 95°C for 45 sec, annealing temperature for 45 sec, 68°C for 60 sec and ending with a seven-minute extension at 68°C. The specificity of products was inspected by agarose gel electrophoresis before they were purified using Agencourt AMPure magnetic beads (Agencourt Bioscience Corporation, Beverly, MA) using the Biomek NX pipetting robot (Beckman Coulter) and eluted in distilled H2O. Sequence PCR was performed using forward or reverse primer with the ABI Prism BigDye™ cycle sequencing Ready Reaction Kit v1.1 (Applied Biosystems). Sequence PCR was run in 10 μl reactions under the following conditions: 96°C for one minute, followed by 25 cycles of 96°C for 10 sec and 50°C for four minutes. Sequencing products were purified using CleanSeq magnetic beads (Agencourt) using the Biomek NX and re-suspended in 10 μl of High Dye formamide (Applied Biosystems). The sequencing products were separated using gel electrophoresis on a 3730 DNA analyzer (Applied Biosystems) and the output data were viewed and analyzed using Sequence Analysis v5.2 (Applied Biosystems) and BiQ Analyzer [45]. One methylated control sample, one unmethylated control and one 50/50 mixture of methylated and unmethylated controls were included in each PCR to ensure that methylated and unmethylated templates were both equally amplified.

### Methylation-specific PCR (MSP)

MSP was used to analyze the promoter region of *DHRS3*. Primer sequences were taken from Furuta et al [17]. The EpiTect MSP kit (Qiagen) was used to amplify methylated and unmethylated templates in separate reactions. The PCR products were separated on a 2% agarose gel with GelRed (Biotium, Hayward, CA) and visualized with UV light. Methylated and unmethylated controls were used to check the specificity of the assay.

### Data analysis

**Figure 4:**
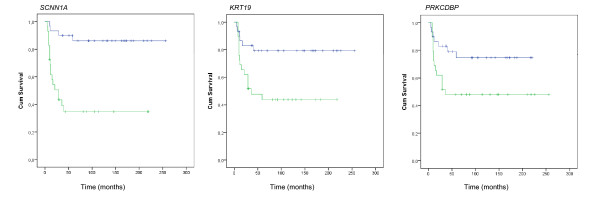
**Survival analysis**. Methylation frequencies of the genes *SCNN1A*, *KRT19 *and *PRKCDBP *analyzed with Kaplan-Meier diagram. Patients are separated by having a methylation frequency above (green line) and below the median (blue line) methylation beta value.

The methylation frequencies of the genes grouped into patients with a 5-year overall survival (OS) versus patients dead of disease, INRG stage, as well as other prognostic factors were compared with Student's two-sided t-test. Considered as prognostic factors; five-year OS, *MYCN *amplification, 1p deletion, 11q deletion, 17q gain, INRG stage, age at diagnosis (cut-off at 18 months) and gene methylation, were analyzed by logistic regression, both as single predictors and in multipredictor models with one gene and one other predictor (Table 4). Kaplan-Meier diagrams were used to illustrate the survival of patients below and above median methylation (Figure 4).

**Table 4:** Odds ratio for gene methylation, with and without adjustment for other prognostic factors.

		Unadjusted OR	Adjusted OR for other prognostic factor			
**Gene**	**CpG site**		**INRG (M/L)**	**MNA**	**11q-del**	**>18 m**

SCNN1A	cg18738906	3.4**	2.2	3.0**	3.1**	3.1**
PRKCDBP	cg16245261	2.5**	2.4*	2.3*	2.4*	2.9**
KRT19	cg11462865	2.5*	2.5*	2.2*	2.3*	2.1*

OR, odds ratio

## Competing interests

The authors declare that they have no competing interests.

## Authors' contributions

HC initiated the project, performed experimental analyses, data analyses, statistical analyses, drafted the manuscript and coordinated the study. AD performed bisulfite sequencing and TLDA. MN and SN performed statistical analysis. RMS aided in cell treatment experiments. CE processed the Illumina methylation arrays at the core facility. PK provided clinical information. TM co-coordinated the study and co-drafted the manuscript. All the authors reviewed and approved the final manuscript.

## Pre-publication history

The pre-publication history for this paper can be accessed here:

http://www.biomedcentral.com/1471-2407/11/66/prepub

## Supplementary Material

Additional file 1 **Venn diagrams of genes identified in the cell treatment study**. (A) Distribution of the top 100 probes (largest fold change between untreated and treated cell lines), detected as up-regulated in the study. Note that there are not always 100 probes in each group, as there are cases in which more than one probe per gene is identified in the top list. (B) Total number of genes up-regulated by treatment and (C) down-regulated. Click here for file

Additional file 2 **Number of genes affected by treatments in at least one cell line**. Click here for file

Additional file 3 **List of genes upregulated by treatment in the respective cell lines**. Click here for file

Additional file 4 **Number of genes affected by treatment in the cell lines and number of genes with a beta value of more than 0.75 in the respective cell lines**. Click here for file

Additional file 5 **The selected genes analyzed in this study**. Click here for file

Additional file 6 **Fold change in gene expression after treatment with 5-Aza-dC and/or TSA in the real-time RT-PCR and microarray experiment**. Click here for file

Additional file 7 **Fold change in gene expression between various tumor characteristics**. Click here for file

Additional file 8 **Patient data**. Click here for file
