# Supplementary figures and images for: Identification of epigenetically regulated genes that predict patient outcome in neuroblastoma
Source: BMC Cancer. 2011 Feb 11;11:66. doi: 10.1186/1471-2407-11-66 (PMC3045360; doi:10.1186/1471-2407-11-66)

## Slide 1
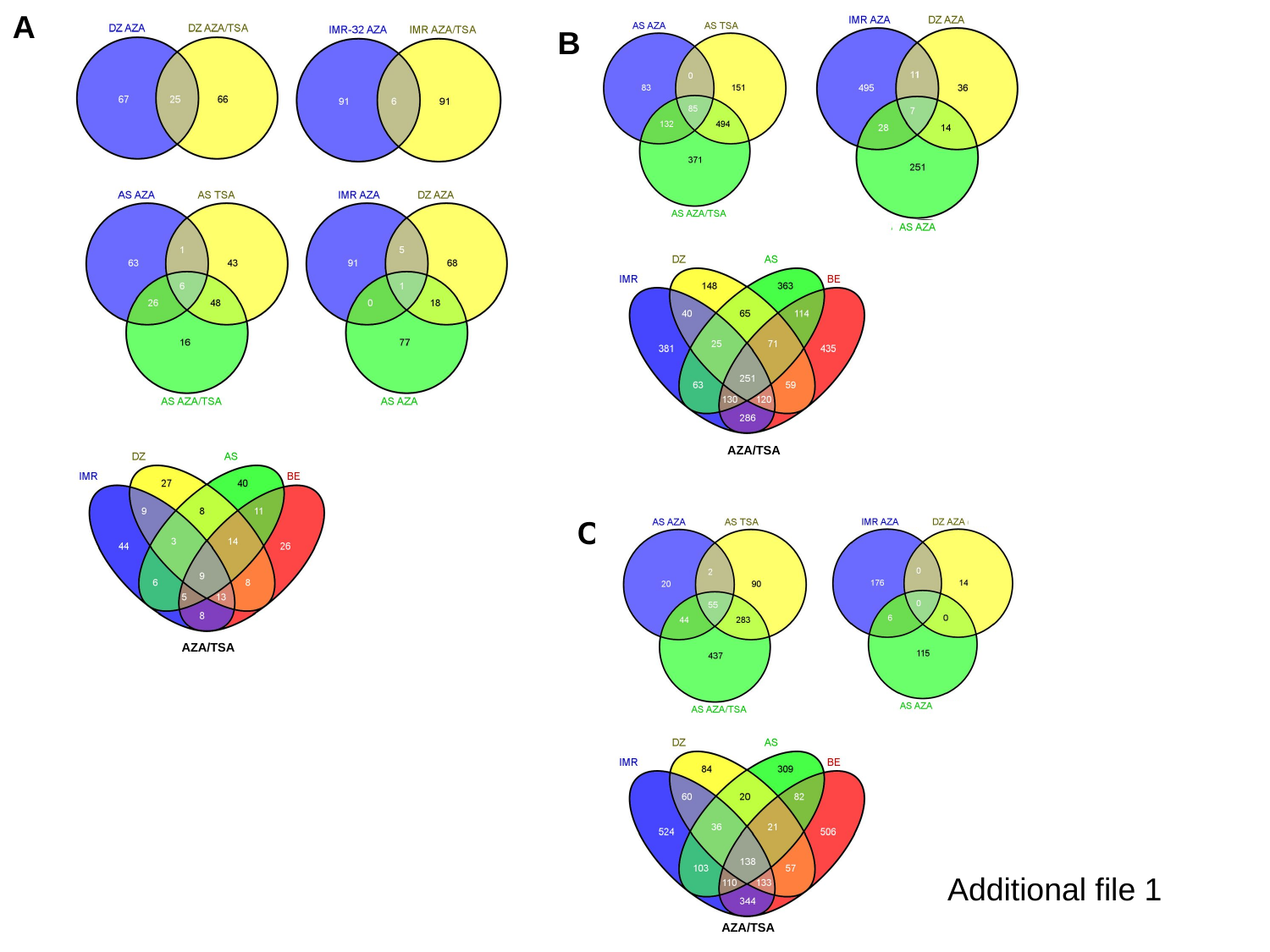

A
B
AZA/TSA
AZA/TSA
C
AZA/TSA
Additional file 1

Supplement: Additional file 1 — Venn diagrams of genes identified in the cell treatment study. (A) Distribution of the top 100 probes (largest fold change between untreated and treated cell lines), detected as up-regulated in the study. Note that there are not always 100 probes in each group, as there are cases in which more than one probe per gene is identified in the top list. (B) Total number of genes up-regulated by treatment and (C) down-regulated. [file 1471-2407-11-66-S1.PPT]
